# Cast off instead of setting anchor: A project to promote housing and work integration for users of integration assistance

**DOI:** 10.1192/j.eurpsy.2025.2175

**Published:** 2025-08-26

**Authors:** J. Krieger, K. Friedrich, C. A. Penkov, V. Rößner-Ruff, W. Wilkening, I. T. Graef-Calliess, M. Ziegenbein

**Affiliations:** 1 Research and Development; 2 Head of the Residential and Day Care Center division, Klinikum Wahrendorff, Sehnde; 3 Medical Director, ZfP Südwürttemberg, Ravensburg; 4 Medical Director, Klinikum Wahrendorff, Sehnde, Germany

## Abstract

**Introduction:**

80% of users of inpatient integration assistance facilities prefer to live in their own home. Access to training opportunities on the labor market is also difficult for people with severe mental illness. Permeability within the care system remains low, meaning that external orientation on the residential or labor market is associated with numerous hurdles. In a dissertation, 18 people from an inpatient integration support facility were followed in a qualitative longitudinal design on their way into their own home.

**Objectives:**

Based on these findings, a project is now being established to support people on their way to greater participation and independence from inpatient facilities. It consists of a multi-stage group program that combines psychoeducational elements with peer-oriented interventions. In addition, an information team of social workers, psychologists and peers is being set up to advise interested users. The project is rounded off by an aftercare program that provides users with advice and is primarily intended to bridge any difficult situations that may arise and build up a support network.

**Methods:**

Users of inpatient integration assistance at a facility in Lower Saxony with an interest in external orientation in the area of living or working. Expected sample size approx. 50 users in a naturalistic longitudinal design. Accompanying evaluation with three measurement points on the development of perceived social support (F-SozU), quality of life (OxCAP-MH) and the subjective benefits of the project to strengthen the participants’ opportunities for participation and the use of further support services.

**Results:**

The project with the individual modules and initial experiences during the implementation phase will be presented.

**Conclusions:**

Possible adjustments to the offer are derived and discussed on the basis of the initial results.

**Disclosure of Interest:**

None Declared

